# Genetic Analysis and Major Quantitative Trait Locus Mapping of Leaf Widths at Different Positions in Multiple Populations

**DOI:** 10.1371/journal.pone.0119095

**Published:** 2015-03-10

**Authors:** Shulei Guo, Lixia Ku, Jianshuang Qi, Zhiqiang Tian, Tuo Han, Liangkun Zhang, Huihui Su, Zhenzhen Ren, Yanhui Chen

**Affiliations:** 1 College of Agronomy, Synergetic Innovation Center of Henan Grain Crops and National Key Laboratory of Wheat and Maize Crop Science, Henan Agricultural University, Zhengzhou, 450002, China; 2 Research Institute of Food Crops, Henan Academy of Agricultural Science, Zhengzhou, Henan, 450002, China; Nanjing Agricultural University, CHINA

## Abstract

**Background:**

Leaf width is an important agricultural trait in maize. Leaf development is dependent on cell proliferation and expansion, and these processes exhibit polarity with respect to the longitudinal and transverse axes of the leaf. However, the molecular mechanism of the genetic control of seed vigor remains unknown in maize, and a better understanding of this mechanism is required.

**Methodology/Principal Findings:**

To reveal the genetic architecture of leaf width, a comprehensive evaluation using four RIL populations was performed, followed by a meta-analysis. Forty-six QTLs associated with the widths of leaves at different positions above the uppermost ear were detected in the four RIL populations in three environments. The individual effects of the QTLs ranged from 4.33% to 18.01% of the observed phenotypic variation, with 14 QTLs showing effects of over 10%. We identified three common QTLs associated with leaf width at all of the examined positions, in addition to one common QTL associated with leaf width at three of the positions and six common QTLs associated with leaf width at two of the positions. The results indicate that leaf width at different leaf positions may be affected by one QTL or several of the same QTLs. Such traits may also be regulated by many different QTLs. Thirty-one of the forty-six initial QTLs were integrated into eight mQTLs through a meta-analysis, and 10 of the 14 initial QTLs presenting an R2>10% were integrated into six mQTLs.

**Conclusions/Significance:**

mQTL1-2, mQTL3-1, mQTL7, and mQTL8 were composed of the initial QTLs showing an R2>10% and included four to six of the initial QTLs that were associated with two to four positions in a single population. Therefore, these four chromosome regions may be hot spots for important QTLs for these traits. Thus, they warrant further studies and may be useful for marker-assisted breeding.

## Introduction

The leaf is the site of the majority of a plant’s photosynthetic activity. Leaf morphological traits, such as size and shape, are major determinants of a plant’s architecture and strongly its affect high-yield performance. Crop leaf photosynthesis plays an important role in dry matter accumulation as well as in grain yield. Breeders and researchers have paid a great deal of attention to leaf shape for ideotype breeding in maize. The flat morphology of a plant leaf is established by the elaboration of two growth processes that determine the extent and direction of growth [1]. Each process shows polarity in regard to the longitudinal and transverse axes of the leaf blade. A leaf blade has three axes: the proximodistal (longitudinal) axis, the central–lateral (transverse) axis, and the adaxial–abaxial axis [2], and polarized cell proliferation and cell expansion along these axes influence the final size and shape of the leaf. Leaf width (central–lateral development) is an important component of leaf morphology. When plants with wide leaves grow side by side, their leaves will eventually overlap and shade each other, leading to shade syndrome in dense plant communities. An appropriate leaf width increases these pronounced shade-avoidance responses and enhances light capture for photosynthesis in dense plantings with a high leaf area index. Therefore, breeding maize with an optimized leaf width for high planting densities is regarded as one of the most important goals for improving maize yields.

Leaf width is a complex quantitative trait, and dissection of these complex traits into their component genetic factors is a prerequisite for their manipulation. Genome mapping using molecular genetic markers offers an excellent opportunity to locate genes or quantitative trait loci (QTLs) that control quantitative characters [3–5]. Several of the QTLs underlying leaf width have been successfully mapped to chromosomes 1, 3, 4, 7, 8, and 9 using two sets of F_2_ populations derived from biparental crosses [6–7]. The contributions of these QTLs to the observed phenotypic variation ranges from 4.86% to 12.60%. Tian et al. [8] identified 34 QTLs associated with leaf width in a genome-wide association study (GWAS) of the Maize Nested Association Mapping (NAM) panel. Although a great deal of research on the genetic basis of leaf width has been reported, the genetic architecture of the trait remains ambiguous. Therefore, further studies on the QTLs underlying the phenotypic variance in this trait are needed.

Leaf width is determined via differentiation along the central–lateral axis of founder cells in the peripheral zone of the shoot apical meristem [2,9,10]. To elucidate the axis-dependent processes of leaf development at cellular and molecular levels, the genes of a series of mutants of *Arabidopsis*, rice, and maize with a specific defect in leaf expansion along the transverse axis were isolated and characterized [11–20]. *Arabidopsis* harbors nine members of the *GROWTH-REGULATING FACTOR* (*GRF*) gene family, most of which are strongly expressed in actively growing and developing tissues [14]. Transgenic plants that overexpress *AtGRF1* and *AtGRF2* exhibit larger leaves and cotyledons, while triple null mutants for *AtGRF1–AtGRF3* display smaller leaves and cotyledons than wild-type plants [15]. The altered leaf growth patterns observed when these genes are overexpressed and in the triple mutants are associated with increases and decreases in the cell size, respectively [14–15]. Similarly, *atgrf5* mutant plants exhibit narrow-leaf phenotypes due to a decreased cell number, while overexpression of *AtGRF5* enhances cell proliferation in the leaf primordium and increases leaf growth [13]. A yeast two-hybrid screen showed that both AtGRF1 and AtGRF5 interact with GIF1. Similar to *grf* mutants, a loss-of-function *gif1* mutant and transgenic RNAi plants develop narrower leaves due to a reduction in the number of cells along the leaf-width axis compared with wild-type plants [14]. In rice, the *NARROW LEAF1* (*NL1*) gene encodes a novel plant-specific protein that regulates polar auxin transport, and an *NL1* mutant was found to present a phenotype of narrow leaves with a reduced number of longitudinal veins [16]. *TDD1* encodes a protein that is homologous to the anthranilate synthase β-subunit, which catalyzes the first step of the Trp biosynthesis pathway and functions upstream of Trp-dependent IAA biosynthesis. The mutant is embryonic lethal, but plants can be regenerated from the calli and produce a phenotype of narrow leaves with reduced small veins [17]. The rolled leaves of *nl7* plants exhibit a markedly decreased width, and their bulliform cells are smaller than those of the wild type [11]. The *NL7* locus encodes a flavin-containing monooxygenase, which shows sequence homology to YUCCA and controls leaf shape via auxin biosynthesis. In maize, the narrow sheath ns1 and ns2 mutants produce extremely narrow leaves due to poor recruitment of the founder cells in the lateral domain region [9], and the narrow and threadlike leaf phenotypes of lbl1 and rgd2 plants are caused by the recruitment of founder cells [12, 20]. These mutants displaying abnormal leaf development are associated with irregular polarity and present narrow leaf lamina. However, these genes only reveal a part of molecular mechanism of leaf width development in crop plants.

Here, we report a novel genetic investigation of maize leaf width based on four sets of recombinant inbred line (RIL) populations and single nucleotide polymorphism (SNP) molecular markers. This study was performed to explore the genetic architecture of maize leaf width to explain the natural variations in leaf width between different lines. Therefore, the leaf widths of various leaves above the uppermost ear were measured, and QTL mapping of the leaf widths was performed.

## Materials and Methods

### Plant materials and field experiments

This study was performed using four sets of RIL populations derived from four crosses between Yu82 and Yu87-1, Yu82 and Shen137, Zong3 and Yu87-1, and Yu537A and Shen137, which are denoted as P1 and P2, P1 and P3, P4 and P2, and P5 and P3, respectively, using a single-seed descent method. The four populations, designated Population 1 (Pop. 1), Population 2 (Pop. 2), Population 3 (Pop. 3), and Population 4 (Pop. 4), included 208, 197, 223, and 212 RILs, respectively, which were used to identify the QTLs associated with leaf width.

The four populations and their corresponding parents were each evaluated in three environments: Pop. 1 was planted in Zhengzhou, Anyang, and Zhumadian, Henan Province, in 2011; Pop. 2 was planted in Shangqiu, Wenxian, and Nanyang, Henan Province, in 2011; Pop. 3 was planted in Zhengzhou, Puyang, and Shangqiu, Henan Province, in 2012; and Pop. 4 was planted in Wenxian, Nanyang, and Anyang, Henan Province, in 2012. All field experiments followed a randomized complete block design using three replicates. Each plot included one row that was 4 m in length and 0.67 m in width, with a total of 17 plants grown at a density of 50,000 plants ha^−1^. The phenotypic data on leaf width were obtained by measuring four consecutive leaves located at different positions above the uppermost ear, and measurements were performed for the following traits: the first leaf width (FirLW), second leaf width (SecLW), third leaf width (ThiLW), and fourth leaf width (ForLW). The leaf width was determined as the typical width across the widest portion of the leaf. The trait values for each family are reported as the averages from five plants from each replicate. The trait measurements averaged over the three experimental environments were used as the preliminary data for the QTL analyses. In addition, the leaf-width values for each position were used in the QTL analysis.

#### Statistical analysis of the phenotypic data

The broad-sense heritability (*h* ^*2*^ *)* of the leaf width at each position above the uppermost ear was calculated according to Knapp et al. [21]. Heritability was calculated as follows: *h* ^*2*^ = *σ*_*g*_^*2*^/(*σ*_*g*_^*2*^ + *σ*_*gy*_^*2*^ */n* + *σ*_*e*_^*2*^ */nr*), where *σ*_*g*_^*2*^ denotes the genetic variance; *σ*_*gy*_^*2*^ denotes the interaction variance between the genotype and the environment; *σ*_*e*_^*2*^ denotes the error variance; *n* denotes the number of environmental effects; and *r* denotes the replication number. *σ*_*g*_^*2*^, *σ*_*gy*_^*2*^ and *σ*_*e*_^*2*^ were estimated through analysis of variance (ANOVA) using the general linear model procedure in SPSS 12.0. The descriptive statistics and simple Pearson correlation coefficients (r) were calculated between the traits using the abovementioned statistical software.

### Construction of molecular linkages and QTL analysis

In total, 3,072 pairs of SNP markers were selected from more than 800,000 SNPs to genotype the 840 RILs (in the four populations) and the five corresponding parents [22]. Among the RILs and between the parents, 1,370, 1,411, 1,479, and 1,371 SNP markers contained polymorphisms in Populations 1, 2, 3 and 4, respectively. Chi-square values were generated for 5,631 SNP markers, and 191, 295, 236, and 269 SNP markers showed significant segregation distortion and failed to be assigned to any linkage groups in Populations 1, 2, 3, and 4, respectively. Genetic linkage maps were constructed using the maximum likelihood mapping function in JoinMap, version 4.0 [23].

The QTL analysis was performed through composite interval mapping (CIM) using WinQTLcart 2.5 [24]. For CIM, Model 6 from the Zmapqtl module was employed to detect the QTLs and their effects, specifying the five markers identified via stepwise regression that explained the majority of the variation for a given trait as the forward and backward parameters, with a window size of 10 cM on either side of the markers flanking the test site [25]. To identify an accurate significance threshold for each trait, an empirical threshold was determined by performing 1,000 random permutations [26]. The QTL position was assigned to the relevant region at the point of the maximum likelihood odds ratio (LOD). The QTL confidence interval was calculated by subtracting one LOD unit from each side from the maximum LOD position [27].

Regarding the additive effects of the QTLs, positive and negative values indicated that alleles from the normal inbred Yu82/Zong3/Yu537A maize line and the inbred Shen137/Yu87-1 maize line, respectively, increased the trait scores. The names of the QTLs were assigned according to the following nomenclature: “q” + “trait abbreviation” + “population code” + “−” + “chromosome number” + “QTL number.”

### QTL integration and meta-analysis

To integrate the QTL information with the measured traits of the four RIL populations, the genetic linkage maps were integrated, and consensus QTLs were identified via meta-analysis [28,29]. The QTLs that mapped to the four RIL populations were projected onto the integrated map using their positions and the confidence intervals shared by the four linkage maps. Some of the controversial markers between the four linkage maps were deleted, which effectively improved the accuracy of the projection.

The meta-analysis was performed using BioMercator2.1 [29]. The Akaike information criterion (AIC) was used to select the QTL model for each chromosome [30]. According to this method, the QTL model with the lowest AIC value is considered to be a significant model for indicating the number of meta-QTLs (mQTLs). The number of mQTLs that showed the best fit to the results in a given linkage group was determined based on a modified Akaike criterion [31]. The names of the mQTLs were assigned according the following nomenclature: “m” + “QTL” + “−” + “chromosome number” + “−” + “meta-QTL number.”

## Results

### Phenotypic performance of leaf width in the five parents and four RIL populations

FirLW, SecLW, ThiLW, and ForLW showed decreased values among the five parental lines and the four RIL populations. Among the five parental lines, the values of all four traits were markedly lower in Yu82, Zong3, and Yu537A than in Yu87-1 and Shen137, with the exception of ForLW, which presented a higher value in Yu537A than in Shen137 in all three environments. Among the RILs, the trait values showed high variability, with transgressive segregation, and the trait values either exceeded or were lower than those of the parental lines. All of the traits exhibited a normal distribution in the four RIL populations (Table 1). The interactions between the genotypes and environment for the measured traits were not significant (data not shown).

**Table 1:** Phenotypic performance of leaf width in five parental lines and four recombinant inbred lines (RILs) across three environments.

Population		FirLW (cm)	SecLW (cm)	ThiLW (cm)	ForLW (cm)
P1 (Yu82)	Mean±S.D.	7.35±0.05	6.80±0.06	6.04±0.05	5.20±0.05
P2 (Yu87-1)	Mean±S.D.	9.40±0.05	8.89±0.05	8.24±0.04	7.35±0.05
P3 (Shen137)	Mean±S.D.	8.73±0.05	7.77±0.04	6.49±0.04	5.40±0.04
P4 (Zong3)	Mean±S.D.	8.21±0.05	6.96±0.04	5.92±0.05	5.23±0.05
P5 (Yu537A)	Mean±S.D.	6.90±0.05	6.59±0.06	6.32±0.05	5.85±0.04
P1 × P2	Mean±S.D.	8.49±0.06	7.95±0.06	7.06±0.06	6.23±0.07
	Range	6.60–10.85	5.96–10.56	5.15–9.76	4.03–8.80
	Skewness	0.38	0.40	0.28	0.31
	Kurtosis	0.44	0.61	0.43	0.32
	*h* ^*2*^ (%)	81.21	79.32	78.88	78.57
	CI	74.46–86.32	75.94–84.67	70.81–84.01	69.97–81.24
P1 × P3	Mean±S.D.	7.43±0.06	6.83±0.05	5.97±0.05	5.3±0.05
	Range	5.39–9.99	4.97–8.56	4.12–7.80	3.70–8.83
	Skewness	0.20	–0.01	0.10	0.87
	Kurtosis	0.21	–0.25	–0.32	0.95
	*h* ^*2*^ (%)	84.33	85.56	79.42	80.67
	CI	73.54–88.67	72.86–90.01	72.52–85.49	71.42–84.58
P4 × P2	Mean±S.D.	7.88±0.07	7.48±0.08	6.6±0.08	6.15±0.1
	Range	5.77–11.10	4.95–20.56	4.20–10.10	3.90–8.72
	Skewness	0.37	0.34	0.44	0.29
	Kurtosis	0.22	0.31	0.51	0.09
	*h* ^*2*^ (%)	79.42	80.54	78.63	81.92
	CI	69.41–85.63	71.75–84.46	70.47–85.22	72.67–87.50
P5 × P3	Mean±S.D.	8.71±0.06	8.41±0.07	7.68±0.07	6.92±0.07
	Range	6.00–11.09	4.47–10.99	5.45–10.29	4.00–9.24
	Skewness	–0.03	–0.24	0.18	0.18
	Kurtosis	0.45	0.49	0.33	0.28
	*h* ^*2*^ (%)	82.44	79.43	75.91	80.63
	CI	68.35–86.87	72.85–85.20	65.84–83.02	71.63–85.07

P1, P2, P3, P4, and P5 represent the inbred lines Yu82, Yu87-1, Shen137, Zong3, and Yu537A, respectively. FirLW, width of the first leaf above the uppermost ear; SecLW, width of the second leaf above the uppermost ear; ThiLW, width of the third leaf above the uppermost ear; ForLW, width of the fourth leaf above the uppermost ear; CI, confidence interval.

The broad-sense heritability of the leaf width at each position above the uppermost ear reached more than 75%, ranging from 75.91% to 85.56% in the four RIL populations (Table 1), and the four measured traits were phenotypically correlated (Table 2). The correlation coefficients between the leaf widths at each position reached significant probability levels at 1%, ranging from 0.62 to 0.93 in the four RIL populations.

**Table 2:** Correlation coefficients for the leaf widths at each position above the uppermost ear in the four RIL populations.

Trait	FirLW	SecLW	ThiLW	ForLW	FirLW	SecLW	ThiLW	ForLW
FirLW		0.93**	0.83**	0.67**		0.93**	0.73**	0.69**
SecLW	0.92**		0.91**	0.76**	0.83**		0.75**	0.78**
ThiLW	0.874**	0.93**		0.81**	0.89**	0.79**		0.62**
ForLW	0.71**	0.76**	0.82**		0.73**	0.65**	0.77**	

FirLW, width of the first leaf above the uppermost ear; SecLW, width of the second leaf above the uppermost ear; ThiLW, width of the third leaf above the uppermost ear; ForLW, width of the fourth leaf above the uppermost ear. The correlation coefficients above the diagonal line in each quadrant of the table are for the Yu82 × Shen137 and Yu537 × Shen137 recombinant inbred lines, and the correlation coefficients below the diagonal line are for the Yu82 × Yu87-1 and Yu82 × Shen137 recombinant inbred lines.

*Significant at P = 0.05;

**significant at P = 0.01.

### SNP data analysis and construction of the genetic linkage map

A total of 3,072 SNP markers were polymorphic among each set of RILs, and 1,370, 1,411, 1,479, and 1,371 were polymorphic between the corresponding Yu82/Yu87-1, Yu82/Shen137, Zong3/Yu87-1, and Yu537A/Shen137 parents, respectively. The percentages of missing genotyping data in the mapping population across the 1,370, 1,411, 1,479, and 1,371 SNP loci were low (1.52%, 1.32%, 1.48%, and 1.68%, respectively). The statistical analyses indicated that the majority of the 5,631 SNP markers followed the expected 1:1 ratio. Ultimately, four genetic linkage maps consisting of all 10 maize chromosomes allocated to 10 linkage groups were constructed based on the 1,179, 1,116, 1,243, and 1,102 SNP markers using JoinMap version 4.0 [30]. The total lengths and average intervals were 1,884.85 cM and 1.59 cM for Yu82/Yu87-1, 1,116 cM and 1.65 cm for Yu82/Shen137, 1,479 cM and 1.50 cM for Zong3/Yu87-1, and 1,102 cM and 1.48 cM for Yu537A/Shen137. The 5,631 SNP loci in the four linkage maps were consistent with the chromosome bin locations in the maps of the Maize Genetics and Genomics Database (MaizeGDB).

### Identification of the QTLs associated with leaf width in the four populations

Forty-six QTLs for FirLW, SecLW, ThiLW, and ForLW were mapped onto all of the maize chromosomes in the four RIL populations, yielding 18 QTLs in Pop. 1, 9 QTLs in Pop. 2, 10 QTLs in Pop. 3, and 9 QTLs in Pop. 4 (Table 3). The individual QTLs explained between 4.33% and 18.01% of the phenotypic variation, while 14 of the QTLs accounted for more than 10% of the phenotypic variation.

**Table 3:** QTLs associated with leaf width in the four recombinant inbred line (RIL) populations across the three environments.

Trait	QTL	Chr	Physical interval (b)	Marker interval	LOD	*R* ^2^ (%)	A	LOD_0.05_
Yu82 × Yu87-1								
FirLW1	qFirLW1-1	1	289251466–291348623	SYN11155–SYN12789	4.58	7.83	0.21	2.75
	qFirLW1-3	3	14227061–16318464	SYN12453–SYN6632	4.37	6.91	–0.20	
	qFirLW1-6-1	6	24530290–37901146	PUT163a181632471246–PZE106015862	3.84	6.34	0.19	
	qFirLW1-6-2	6	158781475–159745835	SYN4194–SYN26189	3.52	6.02	0.18	
	qFirLW1-7	7	144158317–146350574	PZE107094398–PZE107096067	4.44	7.46	–0.21	
	qFirLW1-10	10	128636132–130734664	PZE110072468–PZE110074914	2.74	4.33	–0.16	
SecLW1	qSecLW1-1	1	284313692–288230871	SYN22772–PZE101242961	6.38	12.18	–0.28	2.50
	qSecLW1-2	2	160591358–167053821	PZE102119932–PZE102122951	4.18	7.53	–0.22	
	qSecLW1-3	3	14227061–16318464	SYN12453–SYN6632	8.11	15.21	–0.31	
	qSecLW1-5	5	211446099–211957188	SYN22387—SYN35254	3.64	6.99	–0.21	
	qSecLW1-6	6	158781475–159745835	SYN4194–SYN26189	4.70	8.63	0.23	
ThiLW1	qThiLW1-1	1	284313692–288230871	SYN22772–PZE101242961	5.65	12.65	0.29	3.00
	qThiLW1-3	3	14227061–16318464	SYN12453–SYN6632	4.44	9.50	–0.25	
	qThiLW1-7	7	14764746–22513267	PZE107017355–PZE107022491	4.85	10.30	–0.26	
ForLW1	qForLW1-1	1	284313692–288230871	SYN22772–PZE101242961	5.89	12.13	0.31	2.50
	qForLW1-3	3	14227061–16318464	SYN12453–SYN6632	5.60	11.07	–0.30	
	qForLW1-7	7	13091087–14783643	PZE107015809–PZE107017377	4.02	7.97	–0.25	
	qForLW1-8	8	2039984–4188601	SYN15862–SYN27444	3.51	6.82	0.23	
Yu82 × Shen137								
FirLW2	qFirLW2-1	1	294084520–294310784	PZE101249114–PUT163a760121773730	2.63	6.43	0.21	2.50
	qFirLW2-3	3	20022149–29653189	PZE103027544–PZE103036266	3.06	8.20	–0.20	
	qFirLW2-7	7	14764746–22513267	PZE107017355–PZE107022491	2.87	7.63	–0.23	
SecLW2	qSecLW2-3	3	20022149–29653189	PZE103027544–PZE103036266	3.05	8.16	–0.18	2.75
	qSecLW2-7	7	14764746–22513267	PZE107017355–PZE107022491	3.22	7.97	–0.23	
ThiLW2	qThiLW2-2	2	205138609–213528874	PZE102162330–PZE102173306	2.57	7.19	0.22	2.50
	qThiLW2-7	7	14764746–22513267	PZE107017355–PZE107022491	3.30	9.07	–0.24	
ForLW2	qForLW2-7-1	7	14764746–22513267	PZE107017355–PZE107022491	2.94	8.24	–0.23	2.50
	qForLW2-7-2	7	151735047–153084389	PZE107104709–PZE10710684	2.54	6.68	–0.18	
Zong3 × Yu87-1								
SecLW3	qSecLW3-3	3	21097764–21920219	PZE103028591–PZE103029568	4.11	9.39	0.31	2.50
	qSecLW3-4	4	28985737–22979589	PZE104024889–PZE104021381	4.45	10.32	–0.33	
	qSecLW3-8	8	131124166–128151234	PZE108075601–PZE108074267	2.56	5.62	–0.24	
	qSecLW3-9	9	59199744–55440515	PZE109040189–PZE109038427	4.71	10.73	–0.34	
ThiLW3	qThiLW3-3	3	21097764–21920219	PZE103028591–PZE103029568	3.70	8.87	0.31	2.85
	qThiLW3-8	8	130390943–132374607	SYN14136–PZE108077916	4.87	12.24	–0.36	
	qThiLW3-9	9	59199744–55440515	PZE109040189–PZE109038427	3.80	9.44	–0.33	2.50
ForLW3	qForLW3-3	3	169008365–175554472	PZE103110355–PZE103115618	4.60	18.01	0.42	
	qForLW3-5	5	864121–1249796	SYN12354–SYN20117	2.74	10.14	–0.32	
	qForLW3-8	8	130390943–132374607	SYN14136–PZE108077916	2.93	10.50	–0.32	
Yu537 × Shen137								
FirLW4	qFirLW4-3	3	161590248–166153777	PZE103102501–PZE103104806	2.52	7.22	0.24	2.50
SecLW4	qSecLW4-1	1	87491017–97628380	PZE101095175–PZE101101518	3.09	8.52	–0.30	2.50
	qSecLW4-3	3	161590248–166153777	PZE103102501–PZE103104806	2.64	7.56	0.29	
ThiLW4	qThiLW4-1	1	96547084–97628380	PZE101100960–PZE101101518	3.35	10.04	–0.33	2.50
	qThiLW4-4	4	19723862–22979589	PZE104021381–PZE104019303	2.63	7.74	0.25	
	qThiLW4-8	8	149193811–158103013	PZE108092173–PZE108103951	2.85	8.32	0.26	
ForLW4	q ForLW4-4-1	4	16179673–17653676	PZE109015923–PHM9374.5	3.24	8.98	–0.28	2.75
	q ForLW4-4-2	4	157467388–158138003	PZE-104084296–PUT163a315585431963	5.46	15.14	0.37	
	q ForLW4-9	9	161590248–166153777	PZE103102501–PZE103104806	3.43	11.13	–0.37	

A, additive effect; FirLW, width of the first leaf above the uppermost ear; SecLW, width of the second leaf above the uppermost ear; ThiLW, width of the third leaf above the uppermost ear; ForLW, width of the fourth leaf above the uppermost ear; LOD_0.05_, the genome-wide risk level for a logarithm of odds threshold of P < 0.05.

The 18 QTLs found in Pop. 1 were dispersed across all of the chromosomes, except for chromosomes 4 and 9. Among these QTLs, six were associated with FirLW1, five with SecLW1, three with ThiLW1, and four with ForLW1 (Table 3), and the individual QTLs explained 4.33%–15.21% of the total phenotypic variance. Seven positive alleles among the 18 QTLs were derived from Yu82 and contributed to increased leaf-width values. The QTLs *qFirLW1-3*, *qSecLW1-3*, *qThiLW1-3*, and *qForLW1-3* were all detected within the marker interval SYN12453–SYN6632, and two of these QTLs accounted for more than 10% of the observed phenotypic variation. The QTLs *qSecLW1-1*, *qThiLW1-1*, and *qForLW1-1* were detected within the marker interval SYN22772–PZE101242961, and their contributions to the measured phenotypic variation ranged from 12.13% to 12.65% of the total phenotypic variation. The QTLs *qFirLW1-6-2* and *qSecLW1-1* were detected within the marker interval SYN4194–SYN26189, and the QTLs *qFirLW1-7*, *qThiLW1-7*, and *qForLW1-7* were located in chromosome regions situated close to one another, with one QTL accounting for more than 10% of the phenotypic variation.

The nine QTLs associated with leaf width in Pop. 2 were located on chromosomes 1, 2, 3, and 7, with three of the QTLs being associated with FirLW2, two QTLs with SecLW2, two QTLs with ThirLW2, and two QTLs with ForLW2. The contributions of the QTLs to the observed phenotypic variation ranged from 6.43% to 9.07%. All of the positive alleles with the exception of qFirLW2-1 and qThilLW2-2 were contributed by Yu87-1. The QTLs *qFirLW2-3* and *qSecLW2-3* were located in the region between PZE103027544 and PZE103036266, and the QTLs *qFirLW2-7*, *qSecLW2-3*, and *qThiLW2-7* were located in the region between PZE107017355 and PZE107022491.

The 10 QTLs associated with leaf width in Pop. 3 were found on chromosomes 3, 4, 5, 8, and 9, with 4 QTLs being associated with SecLW3, 3 QTLs with ThiLW3, and 3 QTLs with ForLW3. These QTLs accounted for 5.62%–18.01% of the phenotypic variance. All of the alleles with the exception of qSecLW3-3, qThiLW3-3, and qForLW3-3 were derived from Yu87-1 and contributed to increased trait values. The QTLs *qSecLW3-3* and *qThiLW1-1* were identified in the region between PZE103028591 and PZE103029568. The QTLs *qSecLW3-9* and *qThiLW3-9* were detected in the region between PZE109040189 and PZE109038427 and made contributions of 10.74% and 9.44%, respectively, to the total phenotypic variation. The QTLs *qSecLW3-8*, *qThiLW3-8*, and *qForLW3-8* were located in the chromosome region between SYN14136 and PZE108077916 and accounted for more than 10% of the measured phenotypic variation.

The nine QTLs in Pop. 4 that were associated with leaf width were identified on chromosomes 1, 3, 4, 8, and 9, with one QTL being associated with FirLW4-1, two QTLs with SecLW3, three QTLs with ThiLW3, and three QTLs with ForLW3. These nine QTLs accounted for 7.22%–15.14% of the phenotypic variation. The positive alleles qSecLW4-1, qThiLW4-1, qForLW4-4-1, and qForLW4-9 were derived from Shen137 and contributed to increased leaf-width values. The QTLs *qFirLW4-3* and *qSecLW4-3* were detected in the region between PZE103102501 and PZE103104806. The QTLs *qSecLW4-1* and *qThiLW4-1* were identified in the region between PZE101095175 and PZE101101518 and accounted for 8.52% and 10.04%, respectively, of the total phenotypic variation.

### Genetic map integration and mQTL analysis

To identify the stable and consistent QTLs from the four RIL populations and the pleiotropic or linkage QTLs for the four measured traits, the genetic map and the initial QTLs were integrated via meta-analysis. For all four populations, the maize consensus map contained 2,439 SNP markers and was 1,933.74 cM long, with an average of 0.79 cM between each marker (Table 4). The meta-analysis identified the mQTLs associated with the phenotypic variation in the measured traits. Eight mQTLs were identified from the 46 QTLs that were initially detected based on the variation in leaf width (Table 5). Thirty-one of the 46 initial QTLs (67.39%) were included in these regions.

**Table 4:** Distribution of single nucleotide polymorphism (SNP) markers in the integrated molecular genetic linkage map.

Chromosome	Number of SNP markers	Marker interval range (cM)	Total genetic distance (cM)	Average distance (cM)	Number of integrated QTLs
1	303	0–9.391	258.161	0.852	4
2	248	0–13.139	188.953	0.762	1
3	165	0–10.624	168.003	1.018	11
4	365	0–18.214	291.779	0.799	1
5	244	0.001–10.621	196.211	0.804	2
6	232	0.002–8.048	127.336	0.549	3
7	200	0.003–9.578	202.367	1.012	6
8	370	0–8.146	145.960	0.394	5
9	170	0–11.584	170.532	1.003	2
10	142	0.003–15.656	184.436	1.299	1
Total	2,439		1,933.738	0.793	36

**Table 5:** Meta-QTLs (mQTLs) for leaf-width traits in the four recombinant inbred line (RIL) populations across the three environments.

mQTL	Chr	Flanking marker	Physical interval (b)	No. of QTLs	Integrated QTLs
mQTL1-1	1	PZE101095175–PZE101101518	87491017–97628380	2	qSecLW4-1; qThiLW4-1
mQTL1-2	1	SYN22772–SYN12789	284313692–294310784	5	qFirLW1-1; qSecLW1-1; qThiLW1-1; qForLW1-1; qFirLW2-1
mQTL3-1	3	SYN12453–PZE103036266	14227061–29653189	8	qFirLW1-3; qSecLW1-3; qThiLW1-3; qForLW1-3; qFirLW2-3; qSecLW2-3; qSecLW3-3; qThiLW3-3
mQTL3-2	3	PZE103102501–PZE103104806	161590248–166153777	2	qFirLW4-3; qSecLW4-3
mQTL6	6	SYN4194–SYN26189	158781475–159745835	2	qFirLW1-6-2; qSecLW1-6
mQTL7	7	PZE107015809–PZE107022491	13091087–22513267	6	qThiLW1-7; qForLW1-7; qFirLW2-7; qSecLW2-7; qThiLW2-7; qForLW2-7-1
mQTL8	8	SYN14136–PZE108103951	130390943–158103013	4	qSecLW3-8; qThiLW3-8; qForLW3-8; qThiLW4-8
mQTL9	9	PZE109038427–PZE109040189	55440515–59199744	2	qSecLW3-9; qThiLW3-9

The eight mQTLs were identified on six chromosomes—two on chromosomes 1 and 3, and one each on chromosomes 6, 7, 8, and 9. On average, one mQTL included 3.87 initial QTLs, ranging from two to eight for two to four traits. The initial QTLs included in mQTL3-1 were all identified in three of the populations. It is important to note that 10 (71.43%) of the 14 initial QTLs that showed an *R* ^2^ > 10% in the four populations were included in 6 of the mQTLs: mQTL1-1, mQTL1-2, mQTL3, mQTL7, mQTL8, and mQTL9.

The initial QTLs included in mQTL3-1 were associated with all four leaf-width traits in two populations, while the initial QTLs in mQTL1-2 and mQTL7 were associated with all four traits in one population, and the initial QTLs in mQTL8 were associated with three traits in one population. The QTLs included in the remaining mQTLs were associated with two traits in one population.

## Discussion

Modern maize varieties are more productive than those that were bred only a few decades ago, primarily because of higher population densities and adaptations that permit vigorous growth at high densities. Plant morphologies that enable efficient light interception at high population densities allow increases in yield production under modern field conditions [32–34]. Maize plant morphology is an important and complicated agronomic trait, and current research is focused on leaf width, leaf length, leaf angle, internode elongation, and tassel morphology. One of our interests is investigation of the genetic controls underlying leaf width to improve maize morphology. Only three QTL mapping studies on maize leaf widths have been published to date [6–8], and these studies yielded inconsistent results in regard to the regions of the QTLs. Therefore, further investigations addressing the QTLs underlying the phenotypic variance in leaf width are required. In this study, we identified 46 QTLs associated with leaf width in four RIL populations in three environments: 18 QTLs were identified in Pop. 1; 9 QTLs in Pop. 2; 10 QTLs in Pop. 3; and 9 QTLs in Pop. 4 (Table 3). The individual effects of the QTLs accounted for 4.33% to 18.01% of the observed phenotypic variation, with 14 QTLs contributing over 10%. Thirty-one initial QTLs were combined into 8 mQTLs via meta-analysis, with 10 of the 14 initial QTLs with an *R* ^2^ > 10% being combined into 6 of the mQTLs. These results have important implications for the fine mapping of consensus QTLs.

### Comparison of the mapping results for the four RIL populations

In regard to the widths of the leaves at different positions above the uppermost ear, we identified two common QTLs (qFirLW1-1, qSecLW1-1, qThiLW1-1, qForLW1-1; qFirLW1-3, qSecLW1-3, qThiLW1-3, qForLW1-3) associated with leaf width at all of the positions above the uppermost ear in Pop. 1, which explained 6.91%–15.21% of the phenotypic variation, and one common QTL (qFirLW2-7, qSecLW2-7, qThiLW2-7, qForLW2-7-1) was observed in Pop. 2. One common QTL (qSecLW3-8, qThiLW3-8, qForLW3-8) associated with leaf width at three positions was identified in Pop. 3, which explained 5.62%–10.50% of the observed phenotypic variation. Three common QTLs (qFirLW1-3, qSecLW1-3; qThiLW1-7, qForLW1-7; qFirLW1-6-2; qSecLW1-6) associated with leaf width at two positions were identified in Pop. 1, while two common QTLs (qSecLW3-3, qThiLW3-3; qSecLW3-9, qThiLW3-9) were identified at two positions in Pop. 3, and one common QTL was identified in Pop. 2 and Pop. 4. Additionally, several other QTLs associated with leaf width at one position were identified among the different populations. The results demonstrate that the widths of the leaves at different positions above the uppermost ear can be affected by one QTL or several of the same QTLs. At the same time, the traits may also be regulated by many different QTLs. With respect to the different populations, one QTL between SYN12453 and PZE103036266 was associated with leaf width in at least two positions in three of the populations (Pop. 1, Pop. 2, and Pop. 3), and one QTL between PZE107015809 and PZE107022491 was associated with leaf width in at least two positions in two of the populations (Pop. 1 and Pop. 2). The QTLs that showed high consistency across two or three populations merit further examination using molecular marker-assisted selection (MAS). Although two or three populations were found to exhibit certain similarities because they shared one of the parental lines, several population-specific QTLs were also identified. The population-specific QTLs were attributed to differences in the genetic backgrounds of two populations due to sharing only one common parental line.

In comparison with previous studies examining leaf widths, the present work identified six QTLs that were common across multiple populations or different generations in similar or the same bins as in previous studies. Tian et al. [8] identified 34 QTLs associated with leaf width in a genome-wide association study (GWAS) of the Maize Nested Association Mapping (NAM) panel, which includes six of the QTLs detected in this study. These QTLs comprised alleles between SYN22772 and PZE101242961 on chromosome 1, SYN12453 and PZE103036266 on chromosome 3, PZE-104084296 and PUT163a315585431963 on chromosome 4, PZE-107017355 and PZE-107022491 on chromosome 7, SYN14136 and PZE-108077916 on chromosome 8, and PZE-110072468 and PZE-110074914 on chromosome 10. The results showed that our analyses detected not only stable robust QTLs but also new QTLs, which further indicates that the genetic architecture of leaf width is dominated by small effective alleles. Ku et al. [6, 7] identified 9 QTLs associated with leaf width using two sets of F_2:3_ populations from crosses of Yu82 × Yu87-1 and Yu82 × Shen137. However, three consistent QTLs were identified across two generations (RIL and F_2:3_) from the same populations (Yu82 × Yu87-1, Pop. 1; Yu82 × Shen137, Pop. 2). The two common QTLs between SYN22772 and PZE-101242961 and between PZE-107015809 and PZE-107022491in Pop. 1 and the one common QTL between PZE-103027544 and PZE-103036266 in Pop. 2 found across the RIL and F_2:3_ generations were located in the same regions [6, 7]. This consistency was strongly expected because we use common parents and the same method to detect QTLs, but generation-specific QTLs were still identified. These generation-specific QTLs likely contribute to the different genetic structures and leaf widths observed at different leaf positions, as the respective leaf-width values obtained at four leaf positions above the uppermost ear were used to identify the QTLs in the RIL populations in this study, and the mean values from three positions (the first leaf above the uppermost ear, the leaf of the ear, and the first leaf below the ear) were employed to identify the QTLs in the F_2:3_ populations. These results further confirm that the leaf widths at different leaf positions can be affected by one QTL or a few of the same QTLs or may be regulated simultaneously by many different minor QTLs.

In this study, we identified eight mQTLs from a total of 46 QTLs that were found to be associated with leaf width, which included 71.4% of the QTLs showing an *R* ^2^ over 10%. mQTL3-1 was found to include eight QTLs associated with the leaf width at two positions in one of the three populations and explained 6.51%–15.21% of the observed phenotypic variation. mQTL7 comprised six QTLs associated with leaf width at two positions in one of the two populations and explained 7.61%–10.31% of the phenotypic variation. mQTL1-1 included four QTLs associated with leaf width at all four positions in Pop. 1 and explained 7.46%–12.65% of the measured phenotypic variation. mQTL8 consisted of three QTLs associated with leaf width at three positions in Pop. 3 and explained 5.62%–12.24% of the phenotypic variation. Fine mapping of these four mQTLs is a reliable and feasible strategy for QTL cloning, and these analyses are currently underway in our laboratory.

### Associations between the QTLs and candidate maize genes

Homologous genes associated with leaf width were collected to obtain additional information regarding the genetic architecture of leaf width at different leaf positions in maize. The sequences of the candidate genes from maize, rice, *Arabidopsis*, and other crops were downloaded from NCBI, and the homologous gene sequences from the maize inbred line B73 were identified using MaizeGDB blast, with an E-value cutoff of 10^−10^ and coverage longer than 60%. The candidate genes GRF1, GRF4, and GRF9 were found to be located in mQTL1-2, qThiLW2-2, and qSecLW1-5, respectively. The GRF genes encode a family of putative transcription factors with roles in plant leaf growth [15]. *Arabidopsis* harbors nine members of the GRF gene family, most of which are strongly expressed in actively growing and developing tissues [15]. Molecular genetic analyses of loss-of-function mutants of some of the *Arabidopsis* GRF genes showing narrow-leaf phenotypes [13–15] revealed that these phenotypes were associated not only with reduced cell numbers but also with increased cell size. The effects on both cell proliferation and cell expansion were shown to be regulated by the overexpression of miR396 and may result in the repression of multiple GRF genes in these transgenic plants overexpressing miR396 [35].

In addition, we analyzed the candidate gene without function in 8 mQTL intervals by using Go enrichment analysis (http://geneontology.org/page/go-enrichment-analysis). Among these mQTL intervals, the number of candidate genes without function ranged from 10 to 40 in each mQTL intervals. These genes could be associated with the development of leaf width, and will be needed to be studied further.

### Application of the QTLs associated with leaf width in maize breeding

mQTL1-2, mQTL3-1, mQTL7, and mQTL8 (which contain at least one of the initial QTLs showing an *R* ^2^ > 10%) were identified not only across two generations but also in different populations. They were also detected using the leaf width values obtained for leaves at different positions from the same population. The results indicate that these QTLs are stable major alleles. With the goal of improving leaf morphology, these stable robust QTLs may be very useful for optimizing MAS for maize breeding based on genotypic selection rather than phenotypic performance, which may contribute to enhancing efficiency and reducing breeding time. Therefore, the results of this study may provide valuable information for the further identification and characterization of genes responsible for leaf width in maize.

## References

[1] Tsukaya H (2002) Leaf development In Somerville CR, Meyerowitz EM, editors. The Arabidopsis Book. Rockville, MD: American Society of Plant Biologists.

[2] Bowman JL, Eshed Y, Baum SF (2002) Establishment of polarity in angiosperm lateral organs, Trends Genet. 18: 134–141. 11858837 10.1016/s0168-9525(01)02601-4

[3] Paterson AH, Lander EJ, Hewitt D, Peterson S, Lincoln SE, Tanksley SD (1988) Resolution of quantitative traits into mendelian factors by using a complete linkage map of restriction fragment length polymorphisms, Nature 335:721–726. 2902517 10.1038/335721a0

[4] Lander ES, Botstein D (1989) Mapping Mendelian factors underlying quantitative traits using RFLP linkage maps, Genetics 121: 185–199. 2563713 10.1093/genetics/121.1.185 PMC1203601

[5] Tanksley SD (1993) Mapping polygenes, Ann. Rev. Genet. 27: 205–233. 8122902 10.1146/annurev.ge.27.120193.001225

[6] Ku LX, Zhao WM, Zhang J, Wu LC, Wang CL, Chen YH et al. (2010) Quantitative trait loci mapping of leaf angle and leaf orientation value in maize (Zea mays L.), Theor. Appl. Genet. 10.1007/s00122-010-1364-z 20526576

[7] Ku LX, Zhang J, Guo SL, Liu HY, Zhao RF, Chen YH (2012) Integrated multiple population analysis of leaf architecture traits in maize (Zea mays L.), J. Exp. Bot. 10.1093/jxb/err277 21984652

[8] Tian F, Bradbury PJ, Brown PJ, Hung H., Sun Q, Sherry FG et al (2011) Genome-wide association study of leaf architecture in the maize nested association mapping population, Nat. Genet. 43:159–162. 10.1038/ng.746 21217756

[9] Scanlon MJ (2000) Developmental complexities of simple leaves, Curr. Opin. Plant Biol. 3: 3l–36. 10.1016/s1369-5266(99)00040-0 10679452

[10] Tsiantis M (2001) Control of shoot cell fate: beyond homeoboxes, Plant Cell 13: 733–738. 11283332 10.1105/tpc.13.4.733 PMC526012

[11] Fujino K, Matsuda Y, Ozawa K, Nishimura T, Koshiba T, Fraaije MW et al (2007) NARROW LEAF 7 controls leaf shape mediated by auxin in rice, Mol. Genet. Genomics 279: 499–507. 10.1007/s00438-008-0328-3 18293011

[12] Henderson DC, Muehlbauer GJ, Scanlon MJ (2005) Radial leaves of the maize mutant ragged seedling2 retain dorsiventral anatomy, Dev. Biol. 282: 455–466. 15950610 10.1016/j.ydbio.2005.03.027

[13] Horiguchi G, Kim GT, Tsukaya H (2005) The transcription factor AtGRF5 and the transcription coactivator AN3 regulate cell proliferation in leaf primordial of Arabidopsis thaliana, Plant J. 43: 68–78. 15960617 10.1111/j.1365-313X.2005.02429.x

[14] Kim JH, Kende H (2004) A transcriptional coactivator, AtGIF1, is involved in regulating leaf growth and morphology in Arabidopsis, Proc. Natl. Acad. Sci. USA 101: 13374–13379. 15326298 10.1073/pnas.0405450101 PMC516574

[15] Kim JH, Choi DS, Kende H (2003) The AtGRF family of putative transcription factors is involved in leaf and cotyledon growth in Arabidopsis, Plant J. 36: 94–104. 12974814 10.1046/j.1365-313x.2003.01862.x

[16] Qi J, Qian Q, Bu Q, Li S, Chen Q, Sun J et al (2008) Mutation of the rice NARROW LEAF1 gene, which encodes a novel protein, affects vein patterning and polar auxin transport, Plant Physiol. 147: 1947–1959. 10.1104/pp.108.118778 18562767 PMC2492643

[17] Sazuka T, Kamiya N, Nishimura T, Ohmae K, Sato Y, Imamura K et al (2009) A rice tryptophan deficient dwarf mutant, tdd1, contains a reduced level of indole acetic acid and develops abnormal flowers and organless embryos, Plant J. 60: 227–241. 10.1111/j.1365-313X.2009.03952.x 19682283

[18] Scanlon MJ, Schneeberger RG, Freeling M (1996) The maize mutant narrow sheath fails to establish leaf margin identity in a meristematic domain, Development 122: 1683–1691. 8674408 10.1242/dev.122.6.1683

[19] Scanlon MJ, Chen KD, McKnight CC (2000) The narrow sheath duplicate genes: sectors of dual aneuploidy reveal ancestrally conserved gene functions during maize leaf development, Genetics 155: 1379–1389. 10880496 10.1093/genetics/155.3.1379 PMC1461146

[20] Timmermans MC, Schultes NP, Jankovsky JP, Nelson T (1998) Leafbladeless1 is required for dorsoventrality of lateral organs in maize, Development 125: 2813–2823. 9655804 10.1242/dev.125.15.2813

[21] Knapp SJ, Stroup WW, Ross WM (1985) Exact confidence intervals for heritability on a progeny mean basis. Crop Sci 25:192–194

[22] Ganal MW, Durstewitz G, Polley A, Berard A, Buckler ES, et al (2011) A large maize (Zea mays L.) SNP genotyping array: development and germplasm genotyping, and genetic mapping to compare with the B73 reference genome, PLoS ONE 6: e28334 10.1371/journal.pone.0028334 22174790 PMC3234264

[23] Van Ooijen JW (2006) JoinMap 4. Software for the calculation of genetic linkage maps in experimental populations, B. V. Kyazma, Wageningen, The Netherlands.

[24] Zeng ZB (1994) Precision mapping of quantitative trait loci. Genetics 13: 1457–1468. 8013918 10.1093/genetics/136.4.1457 PMC1205924

[25] Trachsel S, Messmer R, Stamp P, Hund A (2009) Mapping of QTLs for lateral and axial root growth of tropical maize, Theor. Appl. Genet. 119: 1413–1424. 10.1007/s00122-009-1144-9 19760216

[26] Churchill GA, Doerge RW (1994) Empirical threshold values for quantitative trait mapping, Genetics 142: 285–294. 10.1093/genetics/138.3.963 PMC1206241 7851788

[27] Lynch M, Walsh B (1998) Genetics and Analysis of Quantitative Traits, Sinauer Associates, Sunderland, MA.

[28] Arcade A, Falque M, Mangin B, Chardon F, Charcosset A, Joets J (2004) BioMercator: integrating genetic maps and QTL towards discovery of candidate genes, Bioinformatics 20: 2324–2326. 15059820 10.1093/bioinformatics/bth230

[29] Chardon F, Virlon B, Moreau L, Falque M, Joets J, Decousset Murigneux LA et al (2004) Genetic architecture of flowering time in maize as inferred from quantitative trait loci meta-analysis and synteny conservation with rice genome, Genetics 168: 2169–2185. 15611184 10.1534/genetics.104.032375 PMC1448716

[30] Hirotugu A (1974) A new look at the statistical model identification. IEEE Trans. Auto. Contr. 19: 716–723.

[31] Goffinet B, Gerber S (2000) Quantitative trait loci: a meta-analysis. Genetics 155: 463–473. 10790417 10.1093/genetics/155.1.463 PMC1461053

[32] Duvick DN (1977) Genetic rates of gain in hybrid maize yields during the past 40 years, Maydica 22: 187–196.

[33] Duvick DN (1992) Genetic contributions to advances in yield of United States maize. Maydica 37: 69–79.

[34] Duvick DN (2005) The contribution of breeding to yield advances in maize (Zea mays L.), in Advances in Agronomy, vol. 86, Elsevier Academic Press Inc., San Diego, 83–145.

[35] Liu DM, Song Y, Chen ZX, Yu DQ (2009) Ectopic expression of miR396 suppresses GRF target gene expression and alters leaf growth in Arabidopsis, Physiol. Plant. 136: 223–236. 10.1111/j.1399-3054.2009.01229.x 19453503

